# When Worlds Collide: An Interesting Case of Rhinocerebral Mucormycosis Exacerbated by COVID-19 and Diabetic Ketoacidosis Complicated by Intraorbital Hematoma

**DOI:** 10.7759/cureus.21203

**Published:** 2022-01-13

**Authors:** Andrew J Ortega, Sara Alhariri, M Ammar Kalas, Jeff Taclob, Angelica Padilla, Abhizith Deoker

**Affiliations:** 1 Internal Medicine, Texas Tech University Health Sciences Center El Paso, El Paso, USA; 2 Pathology, Texas Tech University Health Sciences Center El Paso, El Paso, USA

**Keywords:** covid-19 associated mucormycosis, intra-orbital hematoma, diabetes type 2, diabetic ketoacidosis (dka), covid 19, rhinocerebral mucormycosis

## Abstract

Mucormycosis is a devastating fungal infection seen in patients who are immunosuppressed or in severe inflammatory states. Mucormycosis has been increasingly seen in the setting of the novel severe acute respiratory syndrome coronavirus 2 (SARS-CoV-2) virus. We describe a 68-year-old male with a past medical history of uncontrolled diabetes mellitus who presented with acute vision loss and was found to have concomitant diabetic ketoacidosis (DKA) and coronavirus disease 2019 (COVID-19) infection on presentation. Rhinocerebral mucormycosis was suspected given the patient's presentation and was confirmed with an ethmoidal sinus biopsy. Our case was further complicated by the presence of cavernous sinus thrombosis, cerebral infarcts, and, later, the development of a left orbital hematoma following therapeutic anticoagulation. This case report aims to address the rare but now increasing incidence of rhinocerebral mucormycosis in the setting of COVID-19, further complicated by DKA, cerebral thrombosis, and intraorbital hematoma.

## Introduction

Mucormycosis is an angioinvasive fungal infection classically seen in immunosuppressed and suboptimally controlled diabetic patients. Mucormycosis is typically caused by *Rhizopus*, *Mucor*, *Rhizomucor*, etc., in the fungi order *Mucorales *[1]. As the term “opportunistic” suggests, there has been an increase in incidence in the setting of the coronavirus disease 2019 (COVID-19) pandemic and commonly in patients with diabetic ketoacidosis (DKA) [2]. Rhinocerebral mucormycosis is deemed the most common type of mucormycosis manifestation [3]. Mucormycosis is typically acquired via inhalation of fungal spores [4]. An enzyme termed ketone reductase is essential for the survival of *Mucorales *as it allows for its viability in acidic environments [4].

We present a case of rhinocerebral mucormycosis in a patient that was synergistically compounded by DKA and COVID-19, further complicated by the development of cavernous sinus thrombosis, embolic brain infarcts, and an intraorbital hematoma. To our knowledge, this is the first documented case of intraorbital hematoma post exenteration in the setting of rhinocerebral mucormycosis.

This case was presented as an abstract at the ACP Texas Chapter Resident Abstract Competition on 11/06/2021.

## Case presentation

A 68-year-old male with a past medical history of type 2 diabetes mellitus and a recent COVID-19 infection (detected at a separate hospital) presented to our institution with a four-day history of left eye pain, left eyelid swelling, progressive vision loss, and headache. These symptoms were preceded by a headache that radiated to the left eye. He was diagnosed with COVID-19 pneumonia at a different hospital approximately 10 days before presentation and was discharged with supportive care instructions as the patient only had mild symptoms and did not require supplemental oxygen. His symptoms at the time included loss of taste, shortness of breath, and a dry cough. His left eye pain and shortness of breath continued to worsen. On the day of presentation, just before arriving at our institution, he had visited a separate medical institution for left eye pain and shortness of breath, where, upon evaluation, he was noted to have an increased left eye intraocular pressure (IOP) elevated at 51 mmHg (high normal is 22 mmHg) and he subsequently underwent canthotomy. He tested positive for COVID-19 and was placed on supplemental oxygen (without further treatment). From that medical facility, he was transferred to our hospital. He denied smoking, alcohol consumption, or use of illicit drugs. His home medications for diabetes mellitus included glipizide and metformin.

On physical examination, he was requiring 15 liters of oxygen through a high-flow nasal cannula and was saturating above 94%. His blood pressure was 125/70 mmHg with a heart rate of 95 beats per minute. Grossly, his left eyelid was swollen with chemosis and proptosis. His IOP bilaterally was normal at 14 mmHg, and his left eye showed lateral canthotomy without cantholysis. Pupils were equal and reactive to light in his right eye, but his left eye revealed a 5 mm fixed pupil to light with complete ophthalmoplegia. He had a loss of V1 sensation with a dull V2 sensation on the left side and no sensory deficit noted on the right side. Cranial nerve examination was otherwise unremarkable. Motor strength was 5/5 in all four extremities, with intact sensation in the rest of his dermatomes. Chest auscultation revealed bilateral crackles. Cardiac examination was unremarkable without any appreciable murmurs auscultated.

Complete blood count revealed reactive thrombocytosis with a platelet level of 562 (562 x 10^9^/L). Complete metabolic profile was significant for elevated creatinine of 1.4 mg/dL (baseline was 0.90 mg/dL), an elevated anion gap metabolic acidosis (23) with a bicarbonate level of 18 mEq/L (normal is less than 12 mEq/L), and hyperglycemia of 379 mg/dL. Beta-hydroxybutyrate was noted to be elevated at 5 mg/dL. Urinalysis was positive for ketonuria. Hemoglobin A1c was elevated at 11% and C-reactive protein (CRP) was greater than 18 mg/dL. Arterial blood gas (ABG) showed a pH of 7.36 with a partial pressure of oxygen (pO2) of 66 mmHg, bicarbonate of 18 mEq/L, and appropriate respiratory compensation with a partial pressure of carbon dioxide (pCO2) of 33 mmHg.

**Figure 1:**
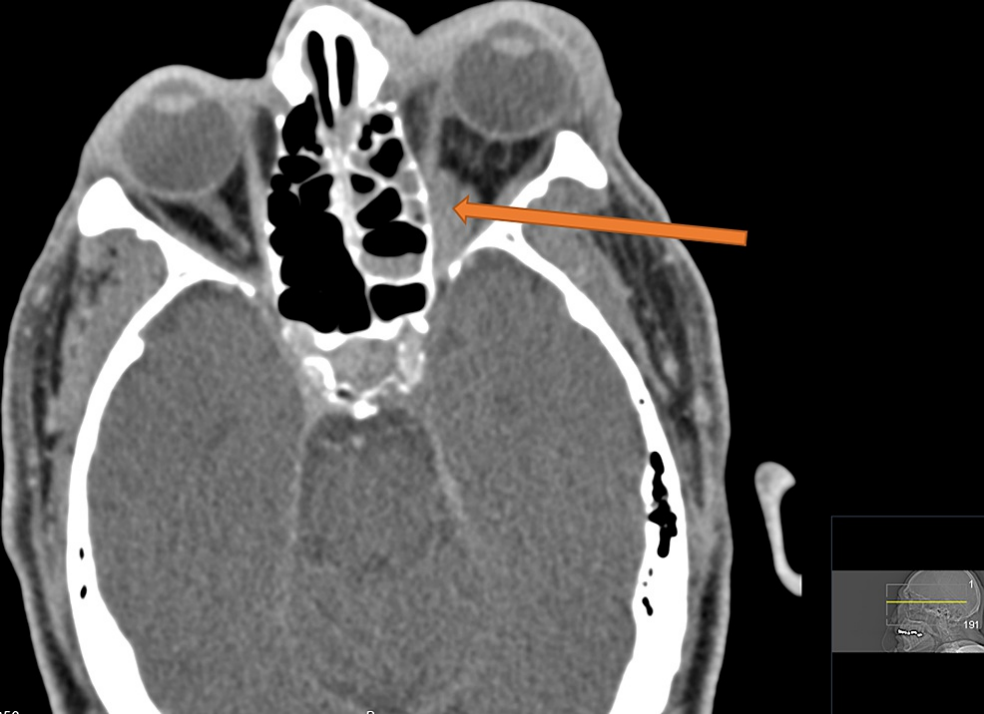
CT of the orbit with contrast. Impression: Left orbital cellulitis likely secondary to extension from sinusitis. There is a 1.7 x 0.7 x 1.4 cm (anteroposterior x transverse x craniocaudal dimensions) hypo-enhancing ill-defined extraconal soft tissue lesion in the posterior inferomedial orbit (orange arrow).

Computed tomography (CT) of the orbit was obtained and demonstrated left orbital cellulitis with mucosal thickening in the left maxillary sinus with a 1.7 x 1.4 cm soft tissue lesion (Figure 1).

**Figure 2:**
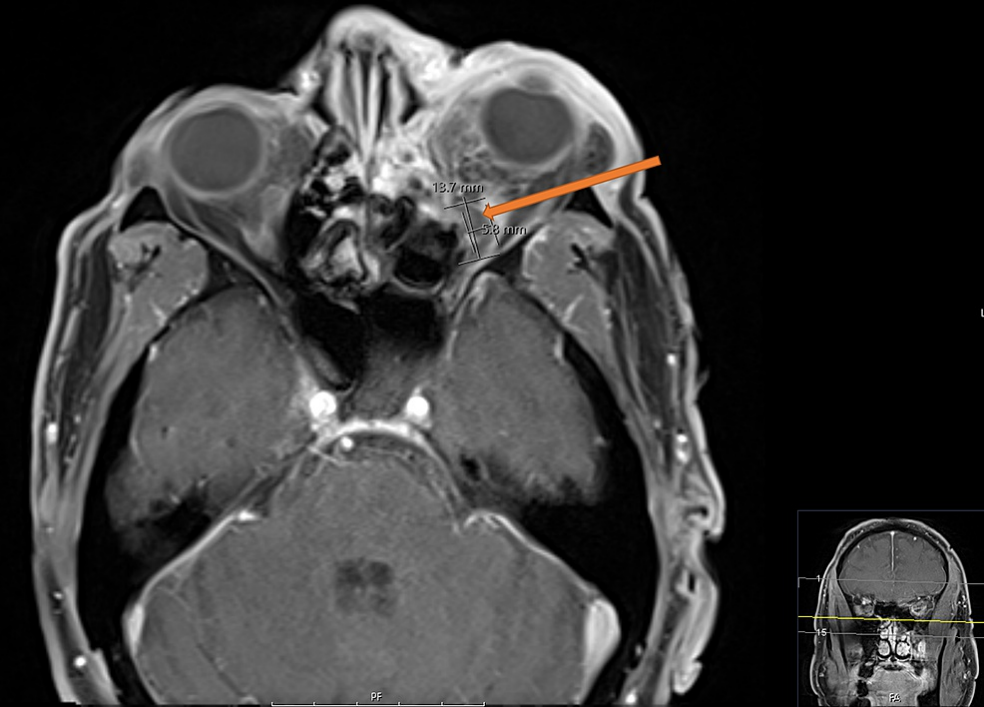
MRI of the face. Impression: Proptotic left globe with left intraorbital cellulitis. There is evidence of a 0.5 x 1.3 x 1.3 cm (transverse, anteroposterior, and craniocaudal dimension) subperiosteal abscess (orange arrow) within the inferomedial aspect of the left orbit secondary to contiguous extension from paranasal sinus disease. There is associated left cavernous sinus thrombosis.

MRI of the brain illustrated a proptotic left globe, and a 0.5 x 1.3 x 1.3 cm left subperiosteal abscess with associated inflammatory neuritis along with embolic infarcts in the left frontal, parietotemporal, and occipital lobes (Figure 2).

Magnetic resonance venography (MRV) confirmed a proptotic left globe along with a left cavernous sinus thrombosis (CST).

The patient was admitted into the ICU and started on a heparin drip for the CST and an insulin drip for his DKA. At this point, ophthalmology, neurology, ENT, and infectious disease (ID) were all involved in his care. Given high suspicion for mucormycosis on initial CT, the patient was started on broad-spectrum antimicrobial coverage with intravenous vancomycin, metronidazole, cefepime, and amphotericin B. As for his acute hypoxic respiratory failure secondary to COVID-19 pneumonia, he was started on dexamethasone and remdesivir. Three days into his hospitalization, his hypoxic respiratory failure acutely decompensated along with worsening facial swelling, necessitating intubation for airway protection.

**Figure 3:**
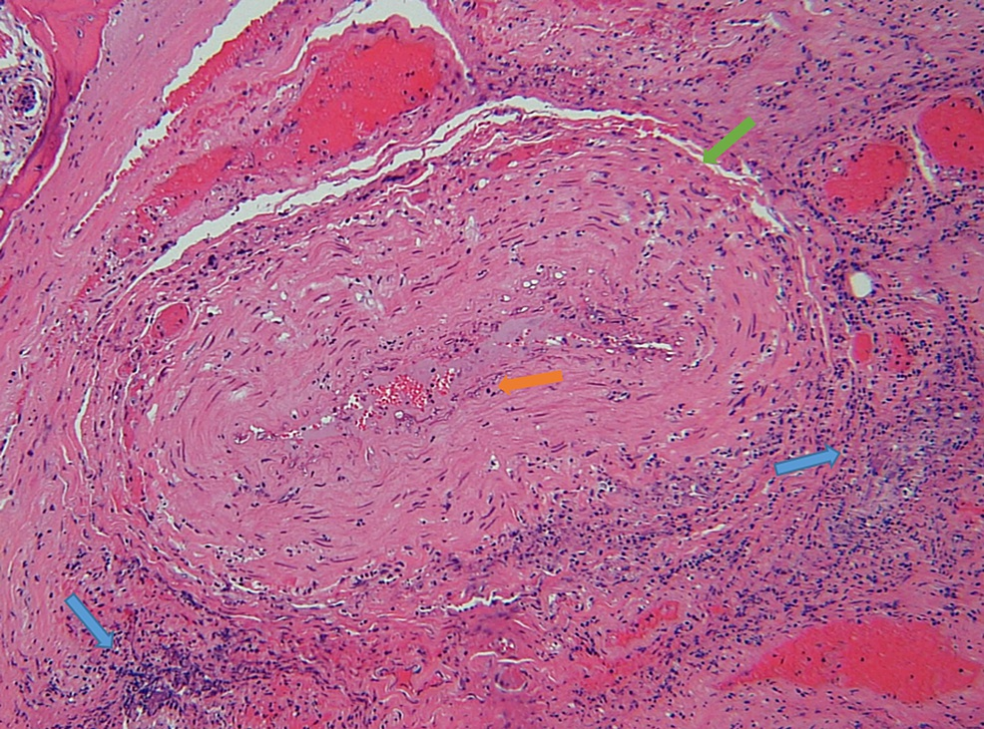
Hematoxylin and eosin (H&E) stain (10x magnification). Histopathology demonstrates angioinvasion of mucormycosis (orange arrow) within the blood vessel (green arrow) with surrounding neutrophilic infiltrate (blue arrow).

**Figure 4:**
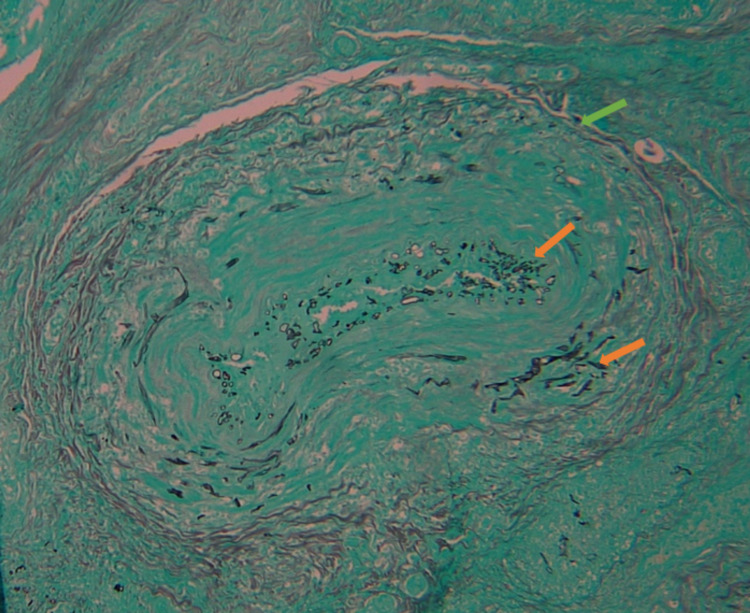
Grocott methenamine silver (GMS) stain (10x magnification). Mucormycosis (orange arrow) is seen within the ethmoidal blood vessel lumen (green arrow).

**Figure 5:**
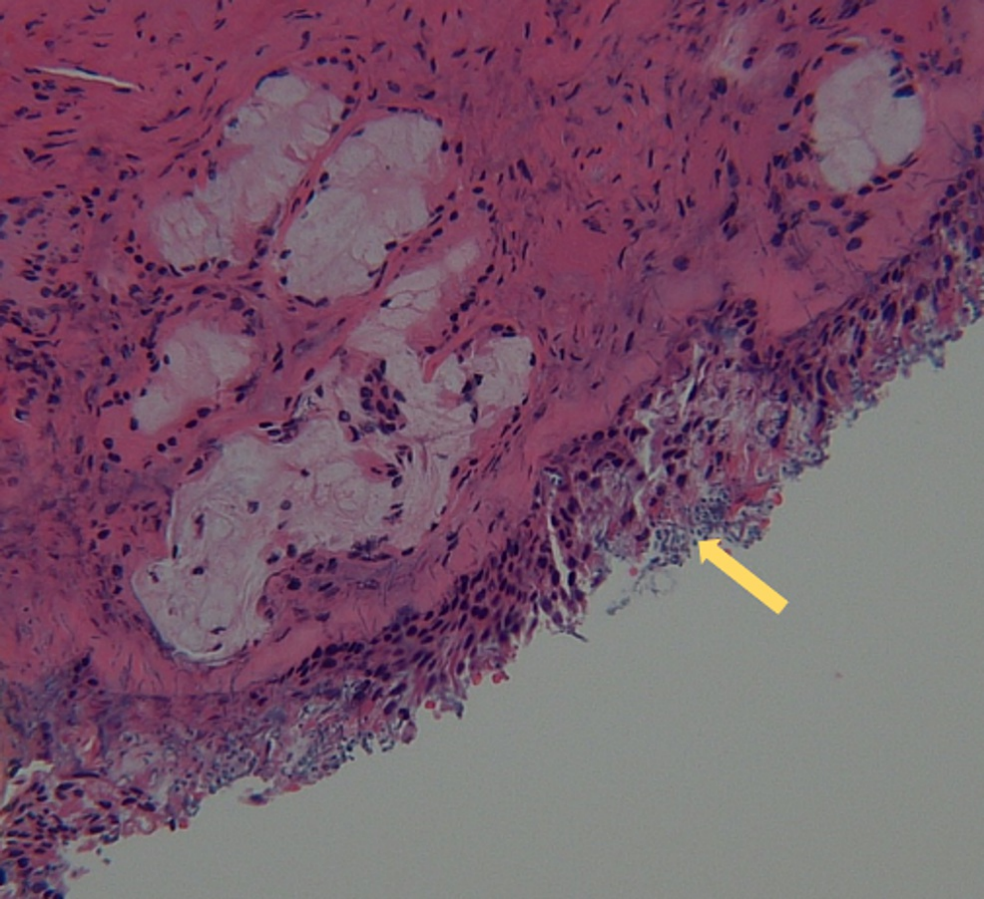
Hematoxylin and eosin (H&E) stain (10x magnification). Histopathology shows *Candida* (yellow arrow) within ethmoidal tissue.

A repeat CT of the head was ordered and was significant for an acute progressive left-sided rhino-orbito-cerebral invasive sinusitis compared to initial CT findings. He subsequently underwent bilateral nasal endoscopy. During endoscopy, the right side was relatively unremarkable. Still, during the evaluation of the left, he was noted to have extensive necrotic tissue and ultimately required debridement from the left side along with sphenoidotomy, ethmoidectomy, and maxillary antrostomy. Debrided tissue from the ethmoidal sinus was evaluated by pathology and was positive for mucormycosis on hematoxylin and eosin (H&E) stain (Figure 3) and Grocott methenamine silver (GMS) stain (Figure 4), and *Candida* on H&E stain (Figure 5).

**Figure 6:**
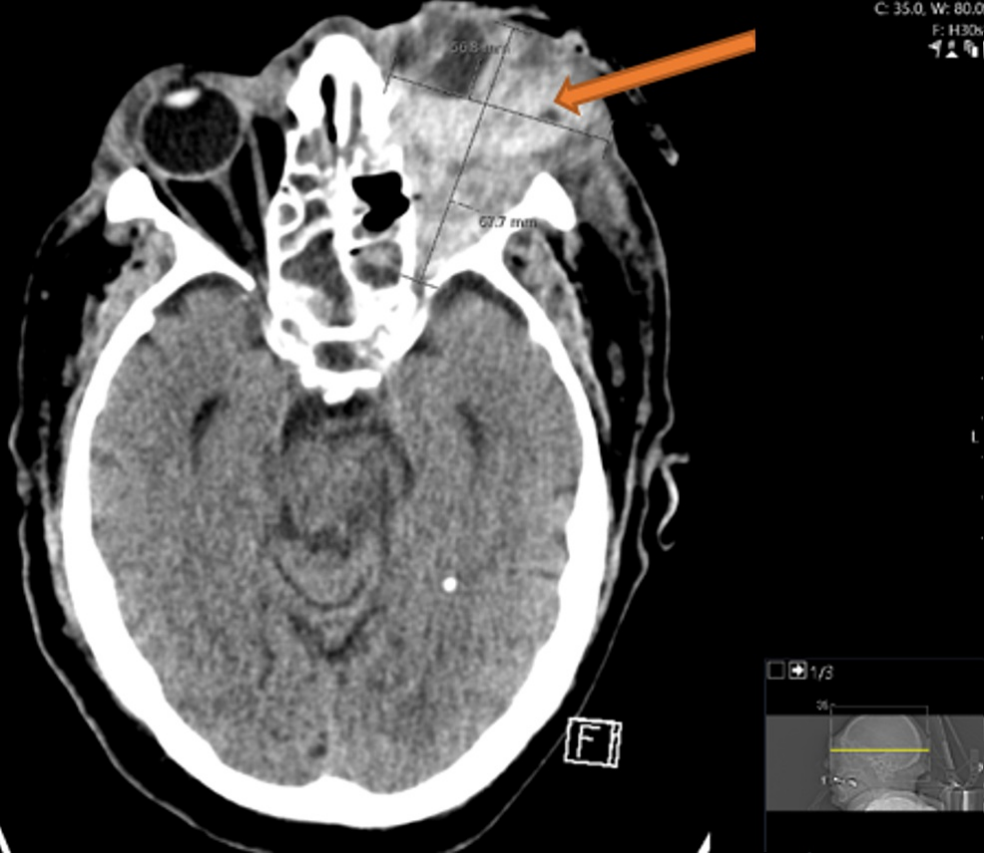
CT of the orbit sella with contrast. Impression: Status post left-sided enucleation with interval development of a large intraorbital hematoma (orange arrow), measuring approximately 67 x 57 mm in maximal anteroposterior and transverse diameter.

Despite the combined endoscopic treatment along with IV amphotericin, there was minimal improvement noted. He was started on a 10-day trial of posaconazole, and he later underwent left orbital exenteration. Despite the orbital exenteration, he continued to deteriorate, requiring three additional nasal endoscopies with debridement involving the inferior turbinates. Approximately one week later, he had involvement of the contralateral orbit. Ten days after the exenteration, he developed a left orbital hematoma confirmed by a CT orbit, measuring 67 x 57 mm (Figure 6).

Heparin was immediately discontinued. Within the next three days, his contralateral right eye became injected, fixed, and non-reactive to light. There was no evidence of a hematoma involving the right eye. Ophthalmology re-evaluated the patient and performed a right retrobulbar injection of 17.5 mg of amphotericin B. Within a month of his exenteration, his left eyelid developed dehiscence, and the orbital wall was surgically removed. The patient remained in the ICU for over one month for continued treatment and stabilization. He had a tracheostomy tube and a percutaneous endoscopic gastrostomy (PEG) tube placed. After stabilization, ID was reconsulted, and the patient was discharged on a two-week course of posaconazole and discharged to a long-term care facility.

## Discussion

Mucormycosis is a deadly angioinvasive fungal infection that has been seen with increased incidence since the COVID-19 pandemic began and is classically associated with DKA and other inflammatory/immunosuppressed states (hematological malignancies, organ transplant, long-term steroid therapy, etc.). The presentation of mucormycosis is variable depending on the infection location (rhinocerebral, cutaneous, pulmonary, gastrointestinal, and dissemination) [5]. *Rhizopus *is the most frequent culprit in diabetic patients diagnosed with mucormycosis [5].

The prevalence of mucormycosis is estimated to be approximately 0.12-0.16 per 10,000 discharges, of which 23% resulted in inpatient mortality. Of note, readmission rates were 37% at the three months mark. This signifies the high morbidity and mortality resulting from mucormycosis [6].

In the COVID-19 era, there are only 101 cases of mucormycosis in patients diagnosed with COVID-19 documented in the world with 80% of those cases noted to be in India and far fewer (nine cases) in the United States [7]. Of note, there are only limited case reports that have been published with mucormycosis (primarily rhino-orbital-cerebral) associated with COVID-19 pneumonia [7]. Multiple mechanisms for this association have been proposed with concomitant DKA infection. Hypoxia secondary to COVID-19 leads to an exploitable environment, which is ideal for the germination of *Rhizopus* spores. Acidosis can be triggered by multiple mechanisms including the lactic acidosis seen with tissue hypoperfusion and sepsis in addition to the respiratory acidosis due to respiratory failure. These synergistic acidic mediums and systemic inflammation in turn cause hyperferritinemia, which provides a source of iron for the further dissemination of mucormycosis [2]. In hyperglycemic states of DKA, the staggering elevation of glucose levels leads to impaired neutrophil function and motility, thus inhibiting the immune system to combat this fungal pathogen [5,7].

**Figure 7:**
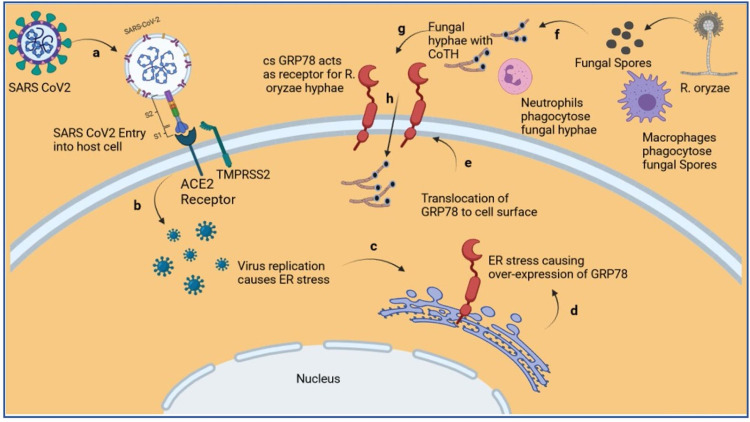
Diagram depicting over-expression and translocation of GRP78 to cell surface due to SARS-CoV-2 replication-induced ER stress, and invasion of hyphae of R. oryzae using cs-GRP78 as its receptor. (a) Entry of SARS-CoV-2 into host utilizing ACE2 and TMRPSS2 as receptors. (b) Replication of SARS-CoV-2 inside the host cell. (c) ER stress-induced due to virus replication. (d) Overexpression of GRP78. (e) Translocation of GRP78 to the cell surface. (f) Germination of *R. oryzae* spores into hyphae after escaping phagocytosis by macrophages. (g) *R. oryzae* hyphae escape phagocytosis by neutrophils and utilize cs-GRP78 receptors (h) to invade the host cell. ACE2: angiotensin-converting enzyme 2; COTH: spore coat protein; cs-GRP78: cell surface GRP78; ER: endoplasmic reticulum; GRP78: glucose-regulated protein 78; *R. oryzae* - *Rhizopus oryzae*; SARS-CoV-2: severe acute respiratory syndrome coronavirus 2; TMPRSS2: transmembrane protease, serine 2. Reprinted from the Journal of Infection and Public Health, Volume 14, Issue 10, Jyotsna Gumashta, Raghvendra Gumashta, COVID19 associated mucormycosis: is GRP78 a possible link? Pages 1351-1357. Copyright 2021. Used with permission from Elsevier [8].

It appears that COVID-19 and mucormycosis also share collaborative mechanisms of cellular uptake via endocytosis. *Mucorales *utilizes a receptor known as glucose-regulated protein 78 (GRP78) as a means of invasion into tissues and endothelial cells [8,9]. This receptor serves as a physiologic receptor in stress and is upregulated in these clinical situations, which are commonly found in the nasal area. COVID-19 classically utilizes the angiotensin-converting enzyme 2 (ACE2) receptors to endocytose within the cell. It has been found that COVID-19 utilizes GRP78 as another means of internalization and intracellular replication [2,8,9]. Given the systemic inflammation caused by DKA, it also can cause an upregulation and synthesis of GRP78 receptors (Figure 7).

Thus, this severe combination of DKA and COVID-19 not only causes a significant upregulation in these receptors but also allows for increased cellular invasion of COVID-19 and *Mucorales *[7,8]. All of these factors provide the optimal medium for *Mucorales *spore germination, which can lead to fatal mucormycosis [5].

The presentation of mucormycosis is variable with a unified general mechanism of vascular invasion leading to necrosis and infarction. Most commonly, mucormycosis presents in the form of rhino-orbital-cerebral mucormycosis, involving the sinuses with a high likelihood of orbital and cerebral invasion. Presenting symptoms generally include fever, purulent discharge, and congestion, and later evolve to eschar formation [8,10]. A close examination should be performed on presentation to assess for signs of orbital involvement such as periorbital swelling, chemosis, proptosis, and, in severe cases, blindness. Facial numbness is common and is secondary to the destruction of the trigeminal nerve branches. Cerebral spread can occur and presents generally with cerebral sinus thrombosis with cranial nerve deficits [10]. In our patient, his rhinocerebral mucormycosis was complicated by CST with embolic brain infarcts.

Moreover, pulmonary involvement can occur with inhalation of the spores and can cause fever with hemoptysis secondary to pulmonary infarcts. In such presentations, patients are at risk of cardiac and mediastinal involvement due to contiguous spread [11]. Gastrointestinal mucormycosis can occur secondary to ingestion of the spores and presents with hematemesis and severe abdominal pain. Other less common presentations include cutaneous mucormycosis, renal mucormycosis, isolated CNS mucormycosis, and, rarely, disseminated mucormycosis.

Prompt diagnostic workup should be done owing to the high morbidity and mortality caused by the disease. High clinical suspicion should be maintained and invasive testing should be included in the initial workup [12]. Cultures generally have a low yield in the diagnosis of mucormycosis due to a high false-negative rate. Invasive testing with biopsy for histopathologic studies is the preferred mode of diagnosis. In cases of rhino-orbital-cerebral mucormycosis, immediate ENT consultation with an endoscopic evaluation of the sinuses should be performed, and specimens should be evaluated for the presence of non-septate broad hyphae. Of note, the absence of hyphae with high clinical suspicion does not rule out the disease and further evaluation should be pursued. In cases of high clinical suspicion with or without suggestive histopathologic findings, an urgent CT scan of the sinuses, orbits, and head should be done to evaluate the extent of the disease and the presence of bony structures involvement [12,13]. MRI is less sensitive in bony structure involvement; however, it should be performed to assess for further extension of mucormycosis [13]. Nevertheless, mucormycosis can be found in healthy patients and, in these cases, their presence in cultures is considered a contaminant in the absence of suggestive clinical signs.

Immediate medical treatment with amphotericin B and surgical debridement are the two mainstays of treating mucormycosis. A delay in treatment of one week nearly doubles the 30-day mortality rate to 66% [14]. Amphotericin B exerts its activity by binding to ergosterol in fungal cell membranes leading to developing pores in the membrane, which leads to leakage of intracellular structures and eventual cellular death [15]. There have been adjunctive treatments with hyperbaric oxygen therapy in addition to local treatment with amphotericin B [14]. In the event that the disease is too advanced, orbital exenteration is the last resort that could leave a patient with disability and disfigurement; however, could be life-saving, as our case depicted [14]. Per Raghav et al., there have been positive outcomes with a clinical cell-based approach with mesenchymal stem cell therapy that have shown promising outcomes with a combination of short-term antifungals, and it is important to note that this therapeutic method assessment is in clinical trials without any conclusive data as of yet [16].

With regards to our case presentation, there are many unique conclusions that can be inferred. Our patient was found to have substantial left-sided nasal necrotic debris that was confirmed to be mucormycosis, only 10 days after his COVID-19 diagnosis. In our case, the patient likely developed CST, four to five days prior to presentation when he began to have symptoms of vision loss, eyelid swelling, and headache. As mucormycosis was the likely triggering factor for his CST, he likely developed the exponential growth of mucormycosis within the first five days of his COVID 19 infection and acute DKA. This highlights that there is a short window (of days) in which complications from mucormycosis can occur. Of note, he was not on any immunosuppressants (dexamethasone) prior to the development of his mucormycosis, but rather, his underlying uncontrolled diabetes alongside COVID-19 pneumonia was the medium through which his mucormycosis developed.

In addition, his hospitalization was such that he was treated with intravenous antifungals with multiple nasal endoscopic debridements. The necrosis of the left side of his sinuses involved the contralateral right side despite treatment with antifungals and debridement. This emphasizes the aggressive and rapidly progressive nature of the disease along with the cascade of multi-factorial triggers.

## Conclusions

Mucormycosis remains a deadly fungal infection that occurs more often in patients with immunocompromised and/or acute inflammatory states. The complex collaborative interplay of DKA and the novel severe acute respiratory syndrome coronavirus 2 (SARS-CoV-2) virus has caused an uprise in mucormycosis cases globally. These inflammatory states, as seen in this report, can contribute to the severity and prognosis of mucormycosis. Prompt recognition and diagnosis of mucormycosis and its associated complications by physicians are of paramount significance. Expeditious administration of antifungal therapeutics along with surgical intervention is crucial due to the high mortality of this pathogen. It should be noted that even in cases where diagnosis and treatment are promptly initiated, morbidity and mortality remain high. Further research targeting diagnostic modalities, anti-microbial development, and surgical approaches is needed to improve patient outcomes.
